# Outcome Predictors of Biopsy-Proven Myeloperoxidase-Anti-Neutrophil Cytoplasmic Antibody-Associated Glomerulonephritis

**DOI:** 10.3389/fimmu.2020.607261

**Published:** 2021-02-04

**Authors:** Yifei Ge, Guang Yang, Xiangbao Yu, Bin Sun, Bo Zhang, Yanggang Yuan, Ming Zeng, Ningning Wang, Huijuan Mao, Changying Xing

**Affiliations:** Department of Nephrology, The First Affiliated Hospital of Nanjing Medical University, Nanjing, China

**Keywords:** myeloperoxidase-anti-neutrophil cytoplasmic antibody-associated glomerulonephritis, vasculitis, histopathologic classification, renal survival, patient survival

## Abstract

**Objective:**

To determine the prognostic values of histopathologic classification of myeloperoxidase-anti-neutrophil cytoplasmic antibody (ANCA)-associated glomerulonephritis and other clinical and laboratory features at the time of presentation on renal and patient survival associated with myeloperoxidase-ANCA-associated glomerulonephritis (MPO-ANCA-GN).

**Methods:**

A total of 112 patients diagnosed with MPO-ANCA-GN from October 2005 to December 2018 were enrolled. The baseline clinical characteristics, renal histopathological data, and risk factors predictive of renal and patient survival were retrospectively analyzed.

**Results:**

All 112 patients underwent renal biopsy. Disease in 32 patients was classified as focal, 26 as mixed, 29 as crescentic, and 25 as sclerotic. Over a median follow-up period of 41.5 months, there were 44 patients dialysis-dependent. The renal survival rate was significantly higher in the focal group than the other groups (*p* < 0.001) and significantly lower in the sclerotic group (*p* < 0.05). In addition, disease histopathologically classified as sclerotic (*p* = 0.044), high serum creatinine level (≥320 μmol/L, *p* < 0.001), low albumin (<30 g/L, *p* = 0.024) and hemoglobin level (<90 g/L, *p* = 0.044) were associated with a greater risk of ESRD. After follow-up, 70 (62.5%) of 112 patients survived. Old age (≥60 years, *p* = 0.018) and low serum albumin (<30 g/L, *p* = 0.006) was significant risk factor for patient survival.

**Conclusion:**

Among patients with MPO-ANCA-GN, those with poor renal function, disease histopathologically classified as sclerotic, and lower albumin and hemoglobin levels were risk factors for ESRD, while older age and low serum albumin level were associated with a greater risk for all-cause mortality.

## Introduction

Anti-neutrophil cytoplasmic antibody (ANCA)-associated vasculitis (AAV) is associated with substantial morbidity and mortality, even with the latest treatment strategies (1). The major clinicopathological variants of AAV include granulomatosis with polyangiitis, microscopic polyangiitis, eosinophilic granulomatosis with polyangiitis, and renal-limited vasculitis (2). Renal involvement in the form of pauci-immune glomerulonephritis is frequently observed in patients with ANCA-associated vasculitis. Kidney disease in AAV is frequently associated with a rapid decline in renal function that persists or continues to worsen without timely and adequate immunosuppressive treatment (3). Moreover, AAV is associated with ANCA specific for myeloperoxidase (MPO-ANCA) or proteinase 3 (PR3-ANCA), depending on geographic and race/ethnic differences. Microscopic polyangiitis accounts for the vast majority of AAV cases, and MPO-ANCA is much more common than PR3-ANCA in China (4–6). Some studies have confirmed that advanced age, renal involvement, lower hemoglobin level, and higher Birmingham Vasculitis Activity Score (BVAS) were predictors of patient outcomes (7, 8), while others report that predictors of renal outcome include histopathologic classification, prior plasmapheresis, and the presence of anemia, as well as serum creatinine and serum albumin levels. A higher degree of proteinuria at diagnosis and during follow-up as well as persistently elevated anti-MPO levels after induction of remission was also predictive of poor renal outcome (9–12). However, relatively few studies have investigated the long-term prognosis of patient and renal survival associated with MPO-ANCA-associated glomerulonephritis (MPO-ANCA-GN). Therefore, the aim of the present retrospective study was to assess the clinical data of MPO-ANCA-GN patients with renal histopathologic findings in order to identify risk factors affecting prognosis.

## Methods

### Ethics Statement 

The study protocol was approved by the Ethics Committee of the First Affiliated Hospital of Nanjing Medical University (Nanjing, Jiangsu, China), and all patients signed a consent form prior to study participation.

### Study Design and Patients

**Figure 1:**
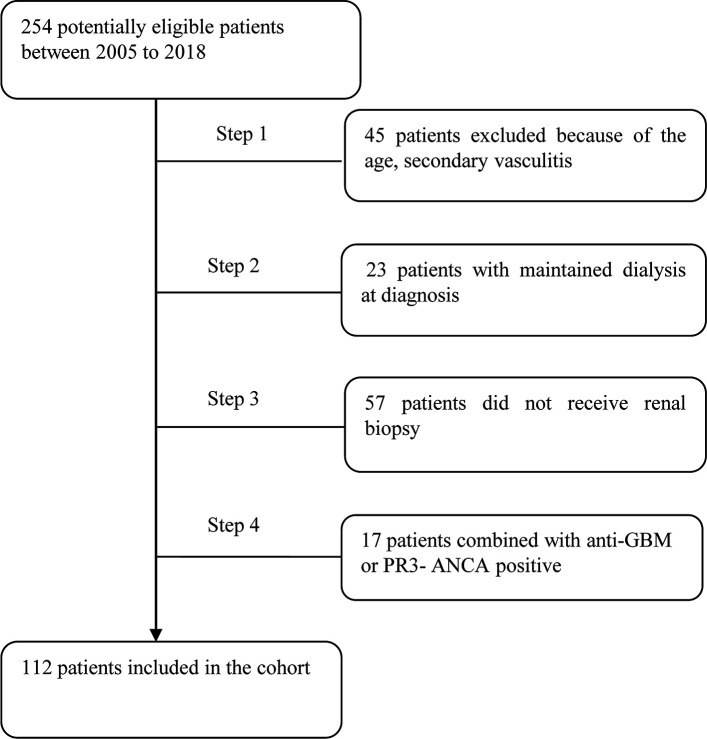
Flow diagram of patients. GBM, glomerular basement membrane.

The cohort of this retrospective study consisted of 112 MPO-ANCA-GN patients newly diagnosed from October 2005 to December 2018 at the First Affiliated Hospital of Nanjing Medical University. Patients who met the following criteria were considered for study inclusion: (1) age of 18–75 years; (2) fulfillment of the Chapel Hill Consensus Conference nomenclature criteria for AAV (2); (3) MPO-ANCA positivity; and (4) diagnosis confirmation by renal biopsy. The follow-up period of the cohort was from October 2006 to December 2019. The exclusion criteria were as follows: (1) PR3-ANCA positivity; (2) secondary vasculitis (*i.e.*, propyl thiouracil-induced AAV, Henoch–Schonlein purpura, lupus vasculitis, cryoglobulinemia, and infection); and (3) the presence of a secondary renal disease, such as anti-glomerular basement membrane disease, immunoglobulin A nephropathy, and diabetic nephropathy. The detail for recruitment was shown in **Figure 1**.

### Clinical and Laboratory Parameters

Baseline data included sex, age, duration of renal involvement, BVAS, urinary levels of red blood cells and 24-h urine protein, as well as serum levels of hemoglobin, albumin, urea, creatinine, uric acid, C reactive protein, and rheumatoid factor (RF). Latex-enhanced turbidimetric immunoassay (Shanghai Ailex Technology Co Ltd) was used for measuring the serum levels of RF according to the manufacturer’s instructions. ANCA levels were measured with antigen-specific enzyme-linked immunosorbent assay (ELISA), according to the manufacturer’s instructions (EUROIMMUN). Vasculitis activity was assessed according to the BVAS, and the estimated glomerular filtration rate (eGFR) was calculated using the Chronic Kidney Disease Epidemiology Collaboration (CKD-EPI) equation (13, 14).

### Renal Histopathology

Renal specimens were examined by light microscopy, immunofluorescence, and electron microscopy using standard procedures. As described by Berden et al., the renal histopathology of ANCA-GN was classified as focal (>50% normal glomeruli), crescentic (>50% glomeruli with cellular crescents), sclerotic (>50% of glomeruli with global sclerosis), or mixed (not characterized by one predominant glomerular phenotype) (15). A semi-quantitative method was used to classify the extent of involvement of tubulointerstitial lesions as mild (score = 1, <25% tubulointerstitial involvement), moderate (score = 2, 25–50% tubulointerstitial involvement), or severe (score = 3, >50% tubulointerstitial involvement). Another method proposed by Brix et al. to evaluate chronic tubulointerstitial lesions score was also adopted by us. The severity of tubular atrophy and interstitial fibrosis (TA/IF) was estimated as the percentage of the affected cortical area (16). All of the renal biopsies were reviewed independently by two experienced renal pathologists blinded to the clinical data. Disagreements were resolved by discussion and consensus. The interobserver variation in histopathologic classification was tested (kappa > 0.8).

### Treatment

During induction therapy, the patient received prednisone (0.5–1 mg/kg/day for 6–8 weeks). Then, the dosage of prednisone was gradually decreased. Induction immunosuppressive agents included MMF 1–1.5 g/day orally, twice monthly intravenous cyclophosphamide at 0.75–1.0 g/m^2^ body surface area. Cases with severe pulmonary hemorrhage or renal histopathology suggestive of crescentic nephritis received intravenous methylprednisolone (500 mg/d for 3 days) or double-filtered plasmapheresis (DFPP). After reaching complete remission or stable partial remission, the patients received maintenance therapy. And the duration of maintenance therapy lasted at least 18 months following successful remission induction. Maintenance therapy included low-dose prednisone combined with CTX (intravenous bolus injection of 0.4–0.6 g every 2 to 3 weeks) or mycophenolate mofetil (MMF). The choice of cyclophosphamide or MMF was dictated by patient preference or cost.

### Follow-Up

Patients were followed up until death, progression to ESRD, or December 31, 2018. ESRD was defined as the requirement of long-term renal replacement therapy (RRT), for example, dialysis or renal transplantation.

### Meta-Analysis

We performed a systematic review of the published literatures. Relevant studies were identified by searching MEDLINE, OVID, SCOPUS, and EMBASE (updated to September 30, 2020) for articles by combinations of the following terms: “ANCA,” “antineutrophil cytoplasmic antibody,” “myeloperoxidase,” “MPO-ANCA,” “vasculitis,” “glomerulonephritis,” “histopathology,” “histopathological,” “kidney,” and “renal”. All eligible articles were retrieved and their references were reviewed to identify additional relevant studies. All studies that validated the histopathological classification of MPO-ANCA-GN patients (that is, MPO-positive patients account for more than 90% of all AAV patients), were eligible for the inclusion. Study endpoints include ESRD and RRT.

### Statistical Analyses

All data were tested for normality. Continuous variable data are expressed as the mean ± standard deviation or as the median (25^th^ and 75^th^ percentiles), while categorical variable data are expressed as the percentage. Non-parametric variables were compared using the Mann–Whitney U test. Independent risk factors associated with patient and kidney survival were assessed using Kaplan–Meier survival analysis and multivariate Cox regression model. Results are expressed as hazard ratios (HRs) with 95% confidence intervals (95% CIs). Analyses were performed using IBM SPSS Statistics for Windows, version 20.0 (IBM Corporation, Armonk, NY, USA). A probability (*p*) value of <0.05 was considered statistically significant.

Meta-analysis was performed using Review Manager Software (RevMan 5.3; The Nordic Cochrane Centre). Hazard ratios (HRs) with their corresponding 95% CIs were used to assess the strength of associations between the histopathological classification and ESRD risk. Heterogeneity across the studies was evaluated by the chi-square-based Q test and quantified using I^2^ tested. The fixed-effects model would be adopted when the studies were found to be homogeneous. Otherwise, the random-effects model would be used. Sensitivity analyses were performed to assess the stability of the results. Publication bias was estimated with Begg's funnel plot and Egger's linear regression test. The analyses were done with Stata software (version 15.1; StataCorp LP, College Station, TX, USA). All *p* values were obtained by two-sided test.

## Results

### Demographic and Clinical Data

The baseline characteristics of all 112 patients enrolled in this study are presented in **Table 1**. The study cohort consisted of 56 males and 56 females with a median age of 64.0 (53.0, 72.0) years, median BVAS of 18.0 (17.0, 21.0), median urine protein of 1.21 (0.60, 2.65) g/24 h, median serum creatinine level at diagnosis of 324.95 (170.15, 653.73) μmol/L, and median eGFR (CKD-EPI) of 17.68 (7.35, 38.48) ml/min. There was no correlation between serum creatinine and urine protein (r = 0.120, *p* = 0.258).

**Table 1:** Baseline data of 112 patients with MPO-ANCA-GN.

Variances	Value
Male (n, %)	56 (50.0%)
Age (years)	64.0 (53.0, 72.0)
Duration of renal disease (months)	1.5 (1.0, 3.0)
BVAS at diagnosis	18.0 (17.0, 21.0)
Initial RRT (n, %)	47.0 (42.0%)
DFPP (n, %)	23.0 (20.5%)
Serum creatinine (μmol/L)	324.95 (170.15, 653.73)
eGFR (ml/min/1.73m^2^)	17.68 (7.35, 38.48)
CRP (mg/L)	40.70 (13.30, 98.20)
RF (IU/ml)	11.40 (10.10, 41.05)
Serum Albumin (g/L)	28.50 (25.28, 32.05)
Hemoglobin (g/L)	79.50 (69.75, 95.75)
Urine red blood cell (/μL)	188.80 (57.10, 664.10)
urine protein (g/24 h)	1.21 (0.60, 2.65)
Extrarenal involvement (n, %)	
Lung	61 (54.5)
ENT	39 (34.8)
Skin	15 (13.4)
Abdominal	6 (5.4)
Fever	18 (16.1)
Treatment (n, %)	
DFPP	23 (20.5)
GC + CTX	80 (71.4)
GC + MMF	23 (20.5)
GC alone	5 (4.5)
No immunosuppressive therapy	4 (3.6)

Among the 112 patients, 47 (42.0%) received initial RRT and 108 (96.4%) were treated with immunosuppressive medications. However, four patients were not treated with immunosuppression due to contraindications, such as severe infection. Induction therapy included glucocorticoids (GCs) plus either MMF (n = 23) or intravenous CTX (n = 80), or GCs alone (n = 5). Seventy-three patients (65.2%) received pulse intravenous infusion of methylprednisolone. In addition to conventional induction therapy (corticosteroids in combination with CTX), 23 (20.5%) patients received an average of 3.63 DFPP therapies. The cumulative dose of CTX was 5.32 g (1.76, 8.62).

### Clinical and Renal Histopathologic Characteristics

All 112 patients underwent renal biopsy. Disease in 32 (28.57%) patients was classified as focal, 26 (23.21%) as mixed, 29 (25.89%) as crescentic, and 25 (22.32%) as sclerotic. The degree of renal insufficiency at diagnosis was more severe in the mixed, crescentic, and sclerotic groups than in the focal group. Anemia was more severe in the crescentic group than in the focal group. Moreover, urine red blood cell counts were significantly greater in the crescentic and sclerotic groups than in the focal group, while rheumatoid factor levels were greater in the focal group than in the sclerotic group and in the mixed group as compared with the crescentic group. The clinical characteristics of the four groups were compared and shown in **Table 2**. The score of acute tubulointerstitial lesions was significantly lower in the focal group than in the other three groups (*p* < 0.001), while the chronic tubulointerstitial score was significantly higher in the sclerotic group than in the focal group (*p* = 0.011). Not surprisingly, the percentage of normal glomeruli was significantly higher in the focal group than in the other three groups (*p* < 0.001) (**Table 3**).

**Table 2:** Clinical features of different histopathological types of MPO-ANCA-GN.

	Focal (n = 32)	Mixed (n = 26)	Crescentic (n = 29)	Sclerotic (n = 25)	*p*
Male (n, %)	17 (53.1%)	12 (46.2%)	15 (51.7%)	12 (48.0%)	0.95
Age(years)	65.0 (49.5, 71.8)	64.3±10.2	64.0 (53.0, 66.5)	66.0 (55.5, 73.5)	0.676
Duration of renal disease (months)	2.0 (1.0, 3.0)	1.0 (0.95, 3.0)	1.0 (1.0, 2.0)	1.0 (1.0, 4.5)	0.508
BVAS at diagnosis	17.7 ± 3.6	18.0 (18.0, 21.3)	19.1 ± 2.8	18.0 (17.0, 19.0)	0.172
Initial RRT (n, %)	4 (12.5%)	13 (50.0%)^b^	13 (44.8%)^b^	17 (68.0%)^b^	**<0.001**
DFPP (n, %)	2 (6.3%)	6 (23.1%)	7 (24.1%)	8 (32.0%)	0.096
Serum Creatinine (μmol/L)	157.2 (85.5, 229.1)	306.1 (185.2, 666.7)^b^	430.6 (211.3.2, 670.0)^a^	557.5 ± 306.6^a^	**<0.001**
eGFR (mL/min/1.73m^2^)	41.3 (27.8, 94.8)	18.6 (7.2, 34.7)^b^	12.1 (6.6, 28.7)^a^	8.9 (5.4, 17.7)^a^	**<0.001**
CRP (mg/L)	76.4 (16.4, 119.8)	27.9 (14.7, 71.2)	49.9 (14.9, 104.3)	26.8 (5.7, 76.4)	0.098
RF (IU/mL)	29.9 (10.6, 152.5)	12.5 (10.7, 36.7)	11.1 (10.1, 31.9)^c^	10.1 (9.5, 11.0)^a^	**0.004**
Serum Albumin (g/L)	29.7 ± 8.2	29.7 ± 4.3	28.1 ± 4.3	28.3 ± 4.8	0.543
Hemoglobin (g/L)	91.3 ± 19.4	88.0 ± 23.9	76.0 (67.5, 88.0)^b^	79.0 (68.0, 82.0)	**0.034**
Urine red blood cell (/μL)	96.3 (27.0, 207.6)	230.0 (107.7, 1087.8)	358.9 (61.7, 929.7)^b^	297.2 (114.8, 648.7)^b^	**0.014**
urine protein (g/24h)	0.92 (0.58, 1.67)	1.18 (0.56, 4.40)	1.63 (1.05, 2.57)	1.55 (0.44, 2.78)	0.312
Extrarenal involvement (n, %)					
Lung	16 (50.0)	14 (53.8)	17 (58.6)	14 (56.0)	0.922
ENT	7 (21.9)	12 (46.2)	11 (37.9)	9 (36.0)	0.264
Skin	3 (9.4)	5 (19.2)	4 (13.8)	3 (12.0)	0.740
Abdominal	3 (9.4)	2 (7.7)	0	1 (4.0)	0.387
Fever	4 (12.5)	5 (19.2)	3 (10.3)	6 (24.0)	0.500

a Focal versus mixed, crescentic, and sclerotic (p < 0.01).

b Focal versus mixed, crescentic, and sclerotic (p < 0.05).

c Mixed versus crescentic and sclerotic (p < 0.05).

**Table 3:** Features of different histopathological types of MPO-ANCA-GN.

	Focal (n = 32)	Mixed (n = 26)	Crescentic (n = 29)	Sclerotic (n = 66625)	*p*
Total glomeruli	18.9 ± 12.3	17.6 ± 8.6	17.3 ± 7.0	17.0 (11.5, 26.5)	0.877
Normal glomeruli (%)	66.1 (50.0, 76.3)	29.8 ± 12.9^a^	22.5 ± 11.7^a^	11.6 (5.6, 21.7)^a^ ^,^ ^b^	**<0.001**
Global sclerotic glomeruli (%)	4.2 (0, 12.2)	23.3 ± 14.4a	11.8 (0, 27.5)	74.1 ± 12.8a^a^ ^,^ ^b^	**<0.001**
Cellular crescents (%)	9.6 (0, 23.1)	22.8 ± 15.0	58.3 (55.1, 64.7)^a^ ^,^ ^b^	16.7 (7.3, 30.2)^c^	**<0.001**
Acute tubulointerstitial lesions score	2.0 (1.0, 3.0)	3.0 (2.0, 3.0)^a^	3.0 (3.0, 3.0)^a^	3.0 (3.0, 3.0)^a^	**<0.001**
Chronic tubulointerstitial lesions score	1.0 (1.0, 1.0)	1.0 (1.0, 2.0)	1.0 (1.0, 2.0)	2.0 (1.0, 2.0)^a^	**0.011**
Tubular atrophy and Interstitial fibrosis (%)	15.0 (10.0, 20.0)	20.0 (18.8, 25.0)	20.0 (20.0, 25.0)^a^	42.6 ± 12.0^a^ ^,^ ^b^ ^,^ ^c^	**<0.001**

a Focal versus mixed, crescentic, and sclerotic (p < 0.01).

b Mixed versus crescentic and sclerotic (p < 0.01).

c Crescentic versus sclerotic (p < 0.01).

### Predictors of Renal Survival in MPO-ANCA-GN

Of the 47 patients who required initial RRT, only seven (14.9%) discontinued RRT, while the other 40 (85.1%) continued to receive maintenance RRT. Over a median follow-up period of 41.5 (17.50, 83.75) months, there were 47 patients dialysis-dependent at baseline and seven came off dialysis, while four progressed to ESRD to follow-up. The incidence of ESRD was lowest in the focal group (4/32, 12.5%) followed by the crescentic (13/29, 44.8%), mixed (12/26, 46.2%), and sclerotic (15/25, 60.0%) groups (*p* < 0.001). Another renal risk score in ANCA-associated glomerulonephritis proposed by Brix et al. showed that patients with the percentage of normal glomeruli <10%, tubular atrophy and interstitial fibrosis ≥25% or eGFR≤15 ml/min were at greater risk of progression to ESRD (**Table 4**).

**Table 4:** Brix renal risk score and renal survival in patients with MPO-ANCA-GN.

Variable	N(%)	HR	95% CI	*P*	Points
Percentage of normal glomeruli (N)					
N0 >25%	60 (53.6%)	Reference			0
N1 10–25%	34 (30.4%)	3.007	1.510–5.986	0.002	4
N2 <10%	18 (16.1%)	2.276	1.014–5.105	0.046	6
Tubular atrophy + interstitial fibrosis (T)					
T0 ≤25%	77 (68.8%)	Reference			0
T1 >25%	34 (30.4%)	3.412	1.846–6.306	<0.001	2
Renal function at time of diagnosis (eGFR)					
G0 >15 ml/min	62 (55.4%)	Reference			0
G1 ≤15 ml/min	50 (44.6%)	9.630	4.058–22.853	<0.001	3

**Figure 2:**
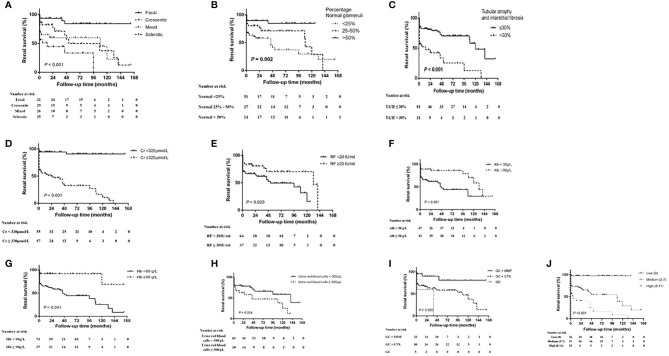
Kaplan–Meier renal survival curve for MPO-ANCA-GN and different risk factors. **(A)** histopathologic classification, **(B)** the percentage of normal glomeruli, **(C)** tubular atrophy and interstitial frosis, **(D)** serum creatinine (Cr) level, **(E)** rheumatoid factor (RF) level, **(F)** serum albumin (Alb) level, **(G)** hemoglobin (Hb) level, **(H)** urine red blood cell count, **(I)** treatment regimen and **(J)** Brix Renal Risk Score according to the risk group (low, 0 points; medium, 2–7 points; high, 8–11 points). CTX, cyclophosphamide; GC, glucocorticoid; MMF, mycophenolate mofetil.

Kaplan–Meier survival analysis showed that the renal survival rate was significantly higher in the focal group as compared with the other three groups (*p* < 0.001, log-rank test) and was significantly lower in the sclerotic group than in the crescentic and mixed groups (*p* < 0.05). Patients with the percentage of normal glomeruli <25% had the highest risk of developing ESRD compared with the patients with the normal glomeruli 25–50% and >50% (*p* = 0.002, log-rank test), and the patients with >50% normal glomeruli had the better renal survival than <25% (*p* = 0.001). The renal survival of patients with TA/IF ≤30% was significantly higher than that with TA/IF >30% (*p <* 0.001, log-rank test). In terms of clinical indicators, high levels of serum creatinine (>320 μmol/L, *p <* 0.001, log-rank test) and urine red blood cells (>300/μl, *p =* 0.04, log-rank test), and low levels of rheumatoid factor (<20 g/L, *p* = 0.020, log-rank test), serum albumin (<30 g/L, *p* = 0.001, log-rank test), and hemoglobin (<90 g/L, *p* < 0.001, log-rank test) were associated with progression to ESRD. Another renal risk score in ANCA-associated glomerulonephritis proposed by Brix et al. showed that the patients with high renal risk score group were more likely to progressed to ESRD than the medium and low score group (*p* < 0.001, log-rank test). In terms of treatment, the renal survival rate of the GCs plus MMF group was significantly higher than that of the GC plus CTX and GCs alone groups (*p* = 0.020, log-rank test). It was worth noting that 20 (25.0%) patients were focal, 24 (30.0%) were crescentic, 19 (23.8%) were mixed and 17 (21.3%) were sclerotic according to renal histopathologic characteristics in CTX group, while patients in the MMF group with nine (39.1%) focal, four (17.4%) crescentic, six (26.1%) mixed and four (17.4%) sclerotic. The percentage of normal glomeruli was 29.91% (16.7%, 50.0%) in CTX group and (33.27 ± 23.27%) in the MMF group. There was no significant difference between the two groups (*p* = 0.818). We also measured the renal function after one month of treatment and found that there was no correlation between the renal function after one month of treatment and renal prognosis for one month. Renal survival curves for the different histopathologic classifications, percentage of normal glomeruli, serum creatinine levels, urine red blood cell counts, rheumatoid factor levels, albumin levels, hemoglobin levels, and treatment regimens are shown in **Figure 2**.

After adjusting for baseline clinical parameters, including age, hemoglobin, urinary protein, serum albumin, creatinine, rheumatoid factor, urine red blood cell counts, treatment regimens, renal histopathological parameters, multivariate Cox regression analysis revealed that histopathological classification of sclerotic disease (*p* = 0.044), serum creatinine levels of ≥320 μmol/L (*p* < 0.001), albumin levels of <30 g/L (*p* = 0.024), and hemoglobin levels of <90 g/L (*p* = 0.044) were significantly associated with a greater risk for progression to ESRD (**Table 5**).

**Table 5:** Cox proportional HR of ESRD (multivariate analysis).

Variable	HR	95% CI	*p*
Albumin, <30 g/L	2.611	1.131– 6.027	0.024
Hemoglobin, <90 g/L	2.958	1.029–8.501	0.044
Creatinine, ≥320 μmol/L	8.644	3.009–24.829	<0.001
Renal histologic class			
Focal group	1.0 (Reference)		
Crescentic group	1.434	0.437–4.705	0.552
Mixed group	2.860	0.896–9.131	0.076
Sclerotic group	3.267	1.031–10.353	0.044

**Figure 3:**
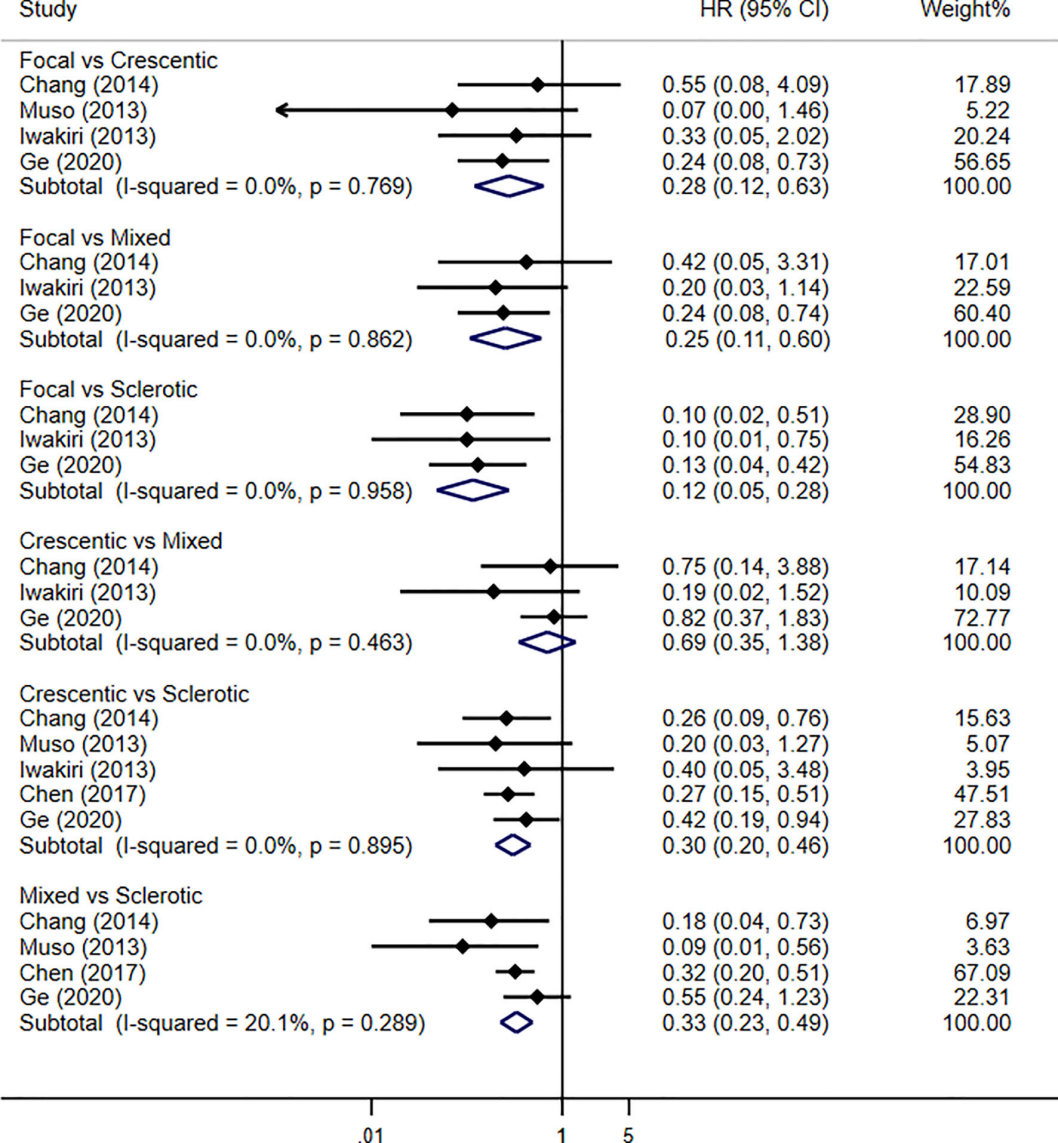
Pooled hazard ratios for renal survival using Berden histopathologic classification in MPO-ANCA-GN patients by traditional meta-analysis.

Four studies were enrolled in our meta-analysis, including our current study. With a total of 637 patients and 197 renal failure events, the renal outcome between focal and crescentic classes showed significant difference in favor of focal class (HR 0.28, 95% CI 0.12–0.63, *p* = 0.002), with no evidence of heterogeneity (I^2^ = 0%, *p* = 0.769). Renal outcome between crescentic and sclerotic classes reported the association of sclerotic class with progression to renal failure (HR 0.30, 95% CI 0.20–0.46, *p* < 0.001), with no evidence of heterogeneity (I^2^ = 0%, *p* = 0.895). For the renal outcome of mixed and sclerotic classes, the results showed the significant difference that was in favor of mixed class (HR 0.33, 95% CI 0.23–0.49, *p* < 0.001), with no evidence of heterogeneity (I^2^ = 20.1%, *p* = 0.289). However, there was no difference in the risk of developing ESRD between the mixed and crescentic classes (HR 0.69, 95% CI 0.35–1.38, *p* = 0.30), with no evidence of heterogeneity (I^2^ = 0%, *p* = 0.463). Renal survival using Berden histopathologic classification in MPO-ANCA-GN patients by traditional meta-analysis is shown in **Figure 3**.

### Risk Factors Influencing Survival of MPO-ANCA-GN Patients

**Figure 4:**
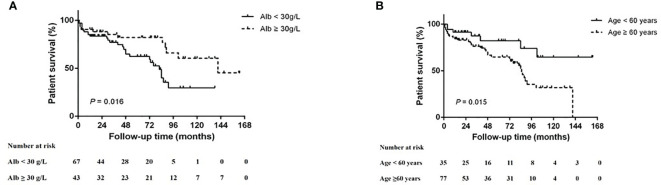
Kaplan–Meier patient survival curve for MPO-ANCA-GN with different risk factors. **(A)** Serum albumin (Alb) level and **(B)** age.

At the end of the follow up, 70 (62.5%) of 112 patients survived (5-year survival rate = 40.6%). Among the non-survival patients, 19 patients died due to severe secondary infection, which was the main cause of death; followed by 12 patients who died of active vasculitis. Deaths in the remaining 11 patients were caused by other therapy-associated adverse events. The 5-year survival rates of the focal, mixed, crescentic, and sclerotic groups were 53.1, 48.1, 27.6, and 32%, respectively (*p* = 0.138). Kaplan–Meier survival analysis suggests that age <60 years (*p* = 0.015, log-rank test) and serum albumin >30 g/L (*p* = 0.016, log-rank test) were associated with better survival (**Figure 4**). After adjusting for baseline clinical parameters, including hemoglobin, creatinine, rheumatoid factor, urine red blood cell counts, treatment regimens, renal histopathological parameters, Cox regression analyses revealed that serum albumin (<30 g/L, *p* = 0.006) was the only significant risk factor for patient survival (**Table 6**).

**Table 6:** Cox proportional HR of patient survival (multivariate analysis).

Variable	HR	95% CI	*P*
Age (≥60 years)	2.818	1.193–6.655	0.018
Albumin, <30 g/L	3.357	1.424–7.915	0.006

## Discussion

The incidence of MPO-AAV is much higher than that of other types of vasculitis in Asian countries, including China and Japan (6, 17, 18). Unlike previous studies that mostly targeted all patients with AAV, only MPO-positive ANCA-GN patients were included in the present study. Hence, the sample size of this study was larger than that of most previous studies. The results of the present study showed that renal survival was highest in the focal group and lowest in the sclerotic group, suggesting a significant correlation between the classification of ANCA-GN and renal prognosis. Similar to some previous studies of ANCA-GN with renal histopathological data, a study of 250 patients with ANCA-GN conducted by Bjørneklett et al. confirmed that renal prognosis was significantly poorer in the sclerotic group than in the focal and combined mixed/crescentic groups (19). In a prospective study of 104 patients with AAV (49 diagnosed with MPO-AAV), renal outcome was best in the focal group, poorest in the sclerotic group, and intermediate in the mixed and crescentic groups. There was no significant difference in renal outcome between the mixed and crescentic groups (20). In another study, after analyzing data from 164 consecutive patients with biopsy-proven renal involvement of ANCA-associated vasculitis, Hilhorst et al. proved that the AGN classification was a practical and informative scheme with which to categorize patients with ANCA-associated vasculitis, but adding the percentage of normal glomeruli to the system seems to improve its predictive value (21). Moreover, in many pathological classification or renal risk scoring tools of ANCA-related vasculitis, such as Berden histopathologic classification and Brix renal risk score, the percentage of normal glomeruli was regarded as one of the key components of the classification. Similar to most previous studies, our study was also found as the predictive value of the percentage of normal glomeruli for renal survival. However, some early or small sample size studies found that histological classification was not predictive of renal prognosis, most studies have confirmed the predictive value of AGN classification for renal prognosis (22, 23). The reason for the differences in the results of the cited studies might be related to the inclusion of only patients with ANCA-GN, that is, both MPO-ANCA-GN and PR3-ANCA-GN. However, the pathology and prognosis of these two types differ. As compared with PR3-ANCA, Quintana et al. found that MPO-ANCA was associated with more severe disease, as demonstrated by a lower frequency of focal disease and a higher frequency of sclerotic disease, as well as more advanced interstitial fibrotic change, a lower glomerular filtration rate at diagnosis, and worse renal function at 1 and 2 years (24). These findings were also confirmed in another study of MPO-ANCA-GN conducted by Chen et al. (9). However, this is still controversial since several studies could not find a certain difference in patient survival in these two types, and one study showed a worse patient survival in PR3-ANCA (25). Moreover, our current study showed that only 15% recovered of 47 patients on RRT, and 44 patients (39.3%) received long-term RRT within 24 months. Additionally, the death rate was 37.5% within a median follow-up of 41.5 months. In contrast, the study by Hilhorst et al. showed 40% recovered, whereas Slot et al. reported that 44% recovered. Also, Slot MC et al. showed only 10% progressed to ESRD (26, 27). The poor renal prognosis of our patients enrolled might be related to the lower eGFR at diagnosis and a higher proportion of patients receiving initial RRT. A study similar to our results reported twenty-three patients (31%) died during a median observation period of 30.5 months from Asian populations (18). Another study of 89 MPO-positive ANCA-GN patients from China showed that 19 (21.3%) patients died on the sixth month (28).

The results of the present study also showed that anemia, hypoalbuminemia, high serum creatinine levels, and a greater concentration of urine red blood cells at diagnosis were related to poor renal survival. In a study of 50 patients with sclerotic ANCA-GN, Menez et al. found that entry eGFR and tubular atrophy were predictors of renal survival (29). Ford et al. also confirmed that decreased renal function was predictive of poor renal outcomes, regardless of the pathological factors identified by renal biopsy (30). Another renal risk score proposed by Brix et al., based on percentage normal glomeruli, percentage tubular atrophy and interstitial fibrosis and eGFR, was considered as a good predictor of renal outcome in ANCA-associated GN. In this study, we also found that Brix renal risk score in ANCA-associated glomerulonephritis was correlated with renal survival. We conducted a meta-analysis of previous studies that included the Berden score and analyzed the relationship between the score and renal prognosis of MPO-ANCA-GN patients and included the current study. We found that our study and most previous studies had similar conclusions. As a previously reported predictor (8, 10), hemoglobin level was also identified in the present study as an important factor influencing renal survival. Different from some previous studies, the results of the present study confirmed that the urine red blood cell count was also predictive of renal survival. Notably, previous studies have rarely mentioned this correlation. A study of 149 patients with AAV conducted by Rhee et al. demonstrated that persistent hematuria was associated with a significantly greater risk of renal relapse, but not persistent proteinuria (31). Similarly, Vandenbussche et al. found that hematuria at remission was a risk factor for renal relapse (32). However, few studies have reported a correlation between hypoalbuminemia and renal survival. For example, Xu et al. analyzed 117 patients with ANCA-associated vasculitis and found that the extent of inflammation was greater, and kidney injury was more severe in patients with hypoalbuminemia than without (33). Coincidentally, another study conducted in China also found that patients with hypoalbuminemia were at greater risk for ESRD (9), which may be due to the fact that MPO-ANCA was the main type of AAV and that albumin had an inhibitory effect on the binding between ANCA and its antigen. In this study, patients receiving GCs plus MMF were at a lower risk of progression to ESRD as compared with those receiving GCs plus CTX and GCs alone, which is consistent with the findings of a previous study (34). However, Tuin et al. found that MMF was more effective than CTX in inducing remission of relapsed ANCA-associated vasculitis (35). The reason for this difference might be that most of the participants their study were PR3-positive and relapsing patients.

In the present study, older age and hypoalbuminemia were significant risk factors for survival of patients with MPO-ANCA-GN. As with most previous studies, the results of the present study also confirmed that age is an important predictor of the survival of patients with ANCA-GN (7, 28, 36). Although rarely mentioned in previous studies, our study confirmed that serum albumin was also a predictor of survival of patients with ANCA-GN. Ahn et al. found that the prognostic nutritional index, which is considered to reflect immune-related nutritional status at diagnosis, might be useful to assess disease severity and predict the prognosis of AAV patients. The prognostic nutritional index is calculated based on the serum albumin level and peripheral blood lymphocyte count (37). In a multicenter retrospective study of 149 patients presenting with ANCA-associated vasculitis and renal involvement, Titeca-Beauport et al. demonstrated that the parameters of disease activity (*i.e.*, albumin level, hemoglobin level, and dialysis status) were significantly associated with 1-year mortality (38). In contrast, other studies found no correlation between serum albumin and patient outcomes (7, 39). In a study involving only patients with MPO-ANCA-GN, the correlation between albumin levels and patient survival was not investigated (9). Hence, the present study is one of relatively few to confirm a correlation between albumin levels and prognosis of patients with MPO-ANCA-GN.

There were several limitations to the present study that should be addressed. First, this was a retrospective single-center study, thus selection bias could not be ruled out. Second, due to the low incidence of PR3-ANCA-GN in Asia, differences in patient outcomes between MPO-ANCA-GN and PR3-ANCA-GN were not investigated. Third, GCs plus CTX treatment was administered to most patients in the present study, thus the choice of treatment may bias the results of renal and patient prognosis.

As one of the few studies focused on MPO-ANCA-GN, the results of the present study confirmed that renal histopathologic classification of ANCA-GN in combination with serum levels of creatinine, albumin, and hemoglobin is associated with renal outcomes. In terms of patient outcomes, age and serum albumin level were confirmed as predictors in this study.

## Data Availability Statement

The original contributions presented in the study are included in the article/supplementary materials; further inquiries can be directed to the corresponding authors.

## Ethics Statement

The studies involving human participants were reviewed and approved by the Ethics Committee of the First Affiliated Hospital of Nanjing Medical University. Written informed consent for participation was not required for this study in accordance with the national legislation and the institutional requirements.

## Author Contributions

Research idea and study design: YG, CX. Data acquisition: GY, XY. Data analysis/interpretation: NW, MZ. Statistical analysis: BS, HM. Supervision or mentorship: BZ, YY. CX takes responsibility that this study has been reported honestly, accurately, and transparently, and accepts accountability for the overall work by ensuring that questions pertaining to the accuracy or integrity of any portion of the work are appropriately investigated and resolved. All authors contributed to the article and approved the submitted version.

## Funding

This work was supported by the Priority Academic Program Development of Jiangsu Higher Education Institutions (grant no. JX10231803).

## Conflict of Interest

The authors declare that the research was conducted in the absence of any commercial or financial relationships that could be construed as a potential conflict of interest.
